# The impact of AI chatbot service quality on consumer purchase intention: the cognitive and affective mediating mechanisms

**DOI:** 10.3389/fpsyg.2026.1931206

**Published:** 2026-09-29

**Authors:** Hongyan Wu, Hewei Li, Yacun Xiao, Yiyue Yao, Yaoxiang Miao

**Affiliations:** 1 School of Humanities and Management, Guilin Medical University, Guilin, China; 2 School of Continuing Education, Guilin Medical University, Guilin, China; 3 Logistics Management Department, The Second Affiliated Hospital of Guilin Medical University, Guilin, China; 4 School of Humanities and Arts, Guangxi Vocational University of Agriculture, Nanning, China

**Keywords:** AI chatbot, e-commerce, perceived usefulness, purchase intention, satisfaction, service quality

## Abstract

**Introduction:**

The increasing prevalence of artificial intelligence chatbots in e-commerce raises questions about how the service quality dimensions of AI chatbots may influence consumer purchase intentions. Drawing on Expectation-Confirmation Theory, the Information Systems Success Model, and the Technology Acceptance Model, this study proposes a conceptual framework to examine these relationships.

**Methods:**

Survey data were collected from 493 Chinese consumers with prior experience using AI chatbots. The study examined perceived personalization, social presence, information quality, and human-AI service continuity as possible antecedents of purchase intention. Perceived usefulness and satisfaction were treated as mediating variables. Partial least squares structural equation modeling was used for the analysis with 5,000 bootstrapped resamples.

**Results:**

Perceived personalization, social presence, information quality, and human-AI service continuity were associated with perceived usefulness. Additionally, perceived personalization, information quality, and human-AI service continuity were positively associated with satisfaction. Conversely, social presence did not show a significant direct effect on satisfaction. Both perceived usefulness and satisfaction are linked to purchase intention, with satisfaction having a stronger direct connection. The mediation analysis revealed that perceived usefulness and satisfaction mediate the relationship between AI chatbot service quality and purchase intention. The only exception was the indirect effect of social presence through satisfaction, which was not statistically significant.

**Discussion:**

This study seeks to contribute to understanding how the service quality of AI chatbots influences consumers’ purchase intentions through perceived usefulness and satisfaction. The results indicate that information quality is the most significant among the service quality dimensions in AI-mediated interactions. The lack of a significant direct impact of social presence on satisfaction suggests that, in utilitarian-oriented e-commerce environments, consumers are more likely to prioritize functional service features over social–emotional factors when assessing AI chatbot services. These findings may inform e-commerce practitioners seeking to improve AI-driven customer service by emphasizing information accuracy and completeness, enhancing personalization, and fostering seamless human-AI service continuity to drive purchase conversions.

## 1 Introduction

Recent advances in artificial intelligence (AI) have significantly reshaped the global retail industry, altering how businesses interact with consumers and create value across digital platforms. AI-driven applications now influence nearly every aspect of retail, including demand forecasting, inventory management, personalized marketing, and customer service automation (Huang and Rust, 2018; Hoyer et al., 2020). AI chatbots have emerged as a transformative innovation, enhancing service efficiency, decreasing operational costs, and enriching customer experiences in the digital commerce (Chen Q. et al., 2023; Shahzad et al., 2024). These technologies leverage advancements in natural language processing, machine learning, and large language models to enable human-like interactions, facilitating real-time engagement with consumers across various channels (Lopes et al., 2024). As a result, AI-powered chatbots have rapidly gained popularity in retail and service sectors. Recent meta-analyses show that factors such as perceived usefulness, ease of use, and service quality influence consumers’ willingness to adopt these technologies (Li B. et al., 2023).

In China’s e-commerce landscape, AI chatbots have become essential to customer service systems. Major platforms, such as Alibaba’s Taobao, JD.com, and Pinduoduo, have implemented advanced chatbots capable of a variety of tasks, including answering product questions, tracking orders, making personalized recommendations, and providing post-purchase support (Jiang K. et al., 2022; Jia et al., 2025). Unlike traditional customer service, which depends on human agents with limited availability, scalability, and consistency, AI chatbots offer distinct advantages, including round-the-clock availability, instantaneous responses, and the ability to handle multiple interactions simultaneously (Ashfaq et al., 2020; Nicolescu and Tudorache, 2022). These features meet the growing consumer demand for immediate, accurate, and personalized service in a highly competitive digital environment (Nguyen et al., 2021). Additionally, integrating AI chatbots with backend enterprise systems enables access to comprehensive customer data, enabling context-aware and predictive interactions that were not possible with conventional methods (Sidlauskiene et al., 2023).

Previous research has contributed significant insights into the factors influencing user satisfaction and continuance intention (Sundjaja et al., 2024; Al-Oraini, 2025), as well as into dialogic interactions and purchase-related behaviors (Jiang H. et al., 2022). It has also explored AI-driven service quality by examining perceived value and need for cognition to predict repurchase intentions (Sharma and Tandon, 2026). However, these studies mainly focus on technology acceptance, post-adoption patronage, and general service evaluations (Guo and Cai, 2024). Consequently, a gap remains regarding how dual cognitive and affective mechanisms, linked to AI chatbot service quality dimensions, influence consumer purchase intentions. Traditional service quality models like SERVQUAL, designed for human-delivered services, may not fully capture how consumers evaluate AI-powered interactions, which involve unique perceptions of humanness, trustworthiness, conversational fluency, and handling complex or emotional exchanges (Lee and Park, 2022; Chen S. et al., 2023; Konya-Baumbach et al., 2023). Recent scholarly efforts have created tailored frameworks for AI chatbot service quality (Chen et al., 2022; Truong and Chen, 2025). Still, empirical evidence on how these service quality facets influence purchase intention through specific cognitive and affective routes remains limited, and there is a need for deeper investigation into the operational dimensions of AI service quality. Addressing this gap offers meaningful theoretical and practical value. Theoretically, it links concepts from the information systems and marketing fields by showing how consumers evaluate AI interactions emotionally and rationally during purchase decisions. This enriches understanding of AI chatbot service quality. Practically, since many e-commerce platforms are investing heavily in AI, clarifying how AI chatbot service quality translates into sales is crucial. Understanding these psychological pathways allows practitioners to better allocate resources, such as whether to focus on improving functional accuracy to boost cognitive utility or enhancing conversational design to increase emotional satisfaction, which can help optimize the return on investment.

To fill this research gap, the study combines three established theoretical frameworks to create a comprehensive model of AI chatbot service quality influences purchase intention. First, Expectation-Confirmation Theory (ECT) (Oliver, 1980) provides a theoretical lens for understanding how users’ initial expectations and post-interaction perceived performance of AI chatbots shape their satisfaction and subsequent behavioral intentions. ECT suggests that satisfaction results from whether experiences confirm or disconfirm expectations, affecting continued use and related behavioral outcomes (Bhattacherjee, 2001). Second, DeLone and McLean’s Information Systems Success Model (ISSM) (DeLone and McLean, 1992, 2003) offers a detailed view of information system quality, including information quality, system quality, and service quality, and their effects on user satisfaction and benefits. This model is pertinent to this study as it provides a validated set of quality dimensions applicable to AI chatbots (Hasan, 2025). Third, the Technology Acceptance Model (TAM) (Davis, 1989) emphasizes perceived usefulness as a key factor influencing technology adoption, consistently shown as a primary predictor across various contexts (Islam et al., 2023; Nguyen et al., 2023). By integrating these frameworks, the study develops a model that considers both cognitive (perceived usefulness) and affective (satisfaction) pathways through which AI service quality impacts purchase intention. While these theories lenses have often used separately to predict technology adoption or post-adoption behaviors, combining them for the e-commerce context provides novel theoretical insights. It shifts focus from mere system use to transaction conversion, showing how specific AI chatbot service quality aspects trigger rational judgments and emotional responses that influence complex financial decisions.

Building on these theoretical foundations, we define the conceptual domain of AI chatbot service quality as the overall assessment of a conversational agent’s functional, relational, affective, and systemic abilities to ensure smooth e-commerce interactions. To operationalize this domain while preventing overlap among sub-dimensions, our inclusion criteria were based on the intersection of the ISSM (DeLone and McLean, 2003) and the unique technological features of AI-mediated communication. Only dimensions that directly reflected the interactive, agent-like nature of AI services were included. As a result, this study identifies and operationalizes four key dimensions of AI chatbot service quality relevant in e-commerce contexts, namely, perceived personalization, social presence, information quality, and human-AI service continuity. Specifically, information quality reflects the core functional output from the ISSM. Perceived personalization addresses the relational and algorithmic capabilities of AI to dynamically adapt services. Social presence captures the affective and conversational features unique to anthropomorphized agents. Human-AI service continuity pertains to systemic integration in hybrid service models. Conversely, exhaustively incorporating all traditional dimensions from models like SERVQUAL or the broader ISSM, such as system reliability, interface aesthetics, or data privacy, would create redundancy and obscure AI-specific features. These factors were excluded as they mainly relate to basic IT infrastructure, general UI design, or risk concerns outside this study’s scope. In addition, recent literature combines AI chatbot service quality dimensions into a single higher-order factor (Leong et al., 2026; Sharma and Tandon, 2026), this research treats them as separate exogenous variables. This approach allows us to analyze the “black box” of AI chatbot service quality, examine the differential effects of each dimension on purchase intention, and identify the primary factors influencing purchase intention.

With regard to the operationalization of these dimensions, perceived personalization refers to the extent to which users perceive that an AI chatbot customizes its responses, recommendations, and interaction style to suit their individual needs, preferences, and behavior patterns (Shen et al., 2024; Panya et al., 2025). This dimension shows an AI system’s ability to use consumer data analytics and machine learning algorithms to provide personalized service experiences (Tan and Liew, 2022). Social presence measures how much users experience psychological proximity, human warmth, and sociability during interactions with AI chatbots (Tsai et al., 2021; Jin and Youn, 2022). This concept, rooted in the media richness theory, reflects the AI chatbot’s capacity to simulate human-like social cues that promote interpersonal connections (Lee et al., 2022). Information quality relates to the accuracy, completeness, timeliness, and reliability of information provided by AI chatbots (DeLone and McLean, 2003; Bhatnagr et al., 2024). As a core function of customer service chatbots, information quality directly influences users’ ability to achieve their transactional goals (Eren, 2021; Leocádio et al., 2024). Human-AI service continuity refers to the smooth integration and cooperation between AI chatbots and human agents, ensuring continuity of service, context retention, and effective escalation of complex issues (Vassilakopoulou et al., 2022; Jeong and Lee, 2025). This dimension recognizes the hybrid service model common in modern e-commerce customer support, where AI and human agents work together to enhance service capabilities (Petrescu and Krishen, 2023; Babar et al., 2025).

This study further proposes that perceived usefulness and satisfaction serve as two mediators that transmit how the four service quality dimensions influence purchase intention. Perceived usefulness, from TAM, captures users’ cognitive evaluation of the functional utility and pragmatic value that AI chatbots offer in aiding e-commerce tasks (Davis, 1989; Huang and Rust, 2020). When users perceive chatbots as effective tools that improve shopping efficiency, reduce decision effort, and deliver timely, relevant information, their assessment of utility increases their likelihood of purchase (Islam et al., 2023; Tan et al., 2025). Satisfaction, grounded in ECT, reflects users’ overall affective and evaluative response to their cumulative experience with AI chatbots (Oliver, 1980; Bhattacherjee, 2001). Satisfied users tend to develop positive attitudes toward the e-commerce platform, perceive less risk, and gain greater confidence in their purchase decisions, leading to higher purchase intention (Belanche et al., 2021; Bilgihan et al., 2025). The dual-mediation framework acknowledges that consumer decisions are affected by both rational evaluations and emotional responses, offering insight into the psychological factors shaping purchase behavior beyond what either factor alone can explain.

## 2 Theoretical background

This study integrates three complementary theories to develop a comprehensive framework for understanding the impact of AI chatbot service quality influences purchase intentions in e-commerce. The ECT, developed by Oliver (1980), is widely applied in information systems research to explain post-adoption satisfaction and continued use. ECT suggests that users’ satisfaction depends on whether their expectations are confirmed or disconfirmed through actual experiences. When expectations are confirmed, users feel satisfied, which boosts their intention to continue using the service (Oliver, 1980; Bhattacherjee, 2001). In AI chatbot contexts, consumers develop initial expectations based on past experiences, marketing messages, and word-of-mouth. If the actual service meets or surpasses these expectations, users experience confirmation, leading to satisfaction and positive behavioral intentions (Ajzen, 1991; Gao et al., 2025). Recent empirical studies support ECT’s relevance for AI chatbots. For example, Ashfaq et al. (2020) found that expectation confirmation significantly influences user satisfaction with AI service agents, which then encourages continued use. Similarly, Gao et al. (2025) showed that AI chatbot problem-solving skills positively influence user confirmation, increasing satisfaction and continued usage intention on e-commerce platforms. These findings underscore ECT’s strength as a framework for studying user reactions to AI-mediated services.

The ISSM, initially introduced by DeLone and McLean (1992) and subsequently refined by DeLone and McLean (2003), forms the second key theoretical foundation of this study. The ISSM identifies six interconnected dimensions of information system success: information quality, system quality, service quality, use, user satisfaction, and net benefits. The model suggests that information quality and system quality directly impact user satisfaction and intended use, which then lead to net benefits for individuals and organizations (DeLone and McLean, 2003; Petter et al., 2013). This study adapts the ISSM to the AI chatbot context, emphasizing service quality dimensions (perceived personalization, social presence, information quality, and human-AI service continuity) as predictors of satisfaction and perceived usefulness, aligning with the original model’s user satisfaction and net benefits components. The ISSM is particularly relevant for this study because it offers a validated framework for understanding how various quality factors shape user outcomes in technology-mediated services (Seddon, 1997; Wirtz et al., 2018). Recent studies on service quality and information quality in AI chatbot research have demonstrated the explanatory power. For example, Chen Q. et al. (2023) used it to examine how AI service quality affects customer loyalty, finding that information quality and service quality significantly influence user satisfaction. Likewise, Hsu and Lin (2023) mentioned that service quality and information quality are key drivers of user satisfaction and loyalty intentions.

The TAM, introduced by Davis (1989), serves as the third key theoretical basis for this study. TAM suggests that the main factors influencing users’ acceptance of information technology are perceived usefulness and perceived ease of use. Perceived usefulness refers to the degree to which a user believes that using a system improves their job performance or task completion, while perceived ease of use assesses the user’s belief that the system is effortless to operate (Davis, 1989; Venkatesh and Davis, 2000). Extensive empirical studies have confirmed TAM’s validity across various technologies, cultures, and user groups, with perceived usefulness consistently emerging as the strongest predictor of the intention to use technology (Alharbi and Alsheehri, 2021). In this study, perceived usefulness is viewed as a vital mediator reflecting users’ cognitive assessment of the functional benefits and utility that AI chatbots offer in supporting their e-commerce activities. This construct aligns with the ‘net benefits’ concept in the ISSM, both emphasizing the practical value gained from interacting with information systems. The decision to focus on perceived usefulness instead of perceived ease of use acknowledges the advancements in AI chatbot technology, where users now rely on intuitive interfaces and prioritize the tangible outcomes of their engagement.

Integrating these three theoretical frameworks provides several key advantages, enhancing both the theoretical foundation and explanatory power of this study. First, ECT offers a theoretical rationale for analyzing satisfaction as a primary outcome variable and mediator, emphasizing the emotional aspect of user experience. Second, the ISSM delivers a thorough framework for defining various service quality dimensions as predictors of user outcomes. Third, TAM adds insight into the cognitive mediator of perceived usefulness, reflecting users’ rational evaluation of technology utility. By combining these perspectives, this study develops a more comprehensive model that explains how both cognitive and affective mechanisms through which AI chatbot service quality affect purchase intention.

## 3 Hypotheses development

### 3.1 Effects of service quality dimensions on perceived usefulness and satisfaction

This study examines how four dimensions of AI chatbot service quality, namely, perceived personalization, social presence, information quality, and human-AI service continuity, influence consumers’ perceived usefulness and satisfaction. Perceived usefulness reflects users’ cognitive judgment of the chatbot’s functional utility, while satisfaction reflects their overall emotional response to the service experience (Bhattacherjee, 2001; Ashfaq et al., 2020). Based on the TAM (Davis, 1989), ECT (Oliver, 1980), and the ISSM (DeLone and McLean, 1992, 2003), it is anticipated that each service quality dimension positively influences both perceived usefulness and satisfaction, albeit potentially through different psychological mechanisms.

Perceived personalization refers to the degree to which users perceive that an AI chatbot understands their individual needs and customizes its responses accordingly (Shen et al., 2024). When AI chatbots provide personalized experiences that meet or surpass user expectations, users experience positive disconfirmation, which boosts satisfaction (Oliver, 1980, 2014). Personalization fosters a sense of being valued and understood as an individual, leading to positive affective responses and greater satisfaction with the service encounter (Lou and Xie, 2020; Shen et al., 2024). It also reduces information overload, simplifies decision-making, and efficiently offers relevant solutions, enhancing users’ perception of the AI chatbot as a useful tool for completing their shopping tasks (Davis, 1989; Kumar and Reinartz, 2016). Empirical research consistently demonstrates that personalized services positively influence customer satisfaction and perceived usefulness in various service environments. (Ahmed and Aziz, 2024; Balaji et al., 2025; Kim and Kim, 2025). Hence, the following hypotheses are proposed:

*H1a*: Perceived personalization positively influences perceived usefulness of AI chatbot services.

*H1b*: Perceived personalization positively influences satisfaction with AI chatbot services.

Social presence refers to the extent to which users feel psychological closeness, human warmth, and sociability during interactions with AI chatbots. The impression of human warmth and personal connection that socially present AI chatbots foster can induce positive emotional responses, boosting overall satisfaction (Deng and Yan, 2025). When users perceive they are engaging with a warm, empathetic, and personable agent rather than a cold, mechanical system, their affective experience improves markedly. Additionally, social presence can reduce the psychological distance between users and technology. It may also increase users’ receptiveness to the information and assistance provided by the AI chatbot (Mende et al., 2019). Moreover, socially present AI chatbots might better grasp user needs through more natural, context-rich conversations, offering more relevant and useful help (Mende et al., 2019; Casaló et al., 2025). Consistent research indicates that higher levels of social presence in tech-mediated interactions correlate with increased user satisfaction and perceived usefulness (Choi et al., 2019; Zhang et al., 2024; Deng and Yan, 2025). Hence, the following hypotheses are proposed.

*H2a*: Social presence positively influences perceived usefulness of AI chatbot services.

*H2b*: Social presence positively influences satisfaction with AI chatbot services.

Information quality pertains to the degree to which information produced by an AI chatbot meets users’ needs regarding accuracy, completeness, timeliness, and reliability (Møller et al., 2025). As outlined in DeLone and McLean’s ISSM, it is a core factor determining system success and user satisfaction (DeLone and McLean, 1992, 2003). When AI chatbot offers accurate, comprehensive, timely, and dependable information, users can perform their shopping tasks more efficiently and make more informed purchasing decisions (Chen Q. et al., 2023; Shahbandi, 2025). Orden-Mejía et al. (2025) mentioned that high-quality information decreases users’ cognitive load and search efforts, directly improving their perception of the AI chatbot is a useful tool. In contrast, providing inaccurate, incomplete, or outdated information disconfirms users’ expectations, resulting in dissatisfaction (Crolic et al., 2021). Szymanski and Hise (2000) demonstrated that high-quality information reflects the service provider’s competence and dedication to customer needs, boosting overall user evaluation of the service. Moreover, empirical studies confirm the positive impact of information quality on perceived usefulness and user satisfaction in various information system contexts (Hsu and Lin, 2023; Møller et al., 2025). Thus, the following hypotheses are proposed:

*H3a*: Information quality positively influences perceived usefulness of AI chatbot services.

*H3b*: Information quality positively influences satisfaction with AI chatbot services.

Human-AI service continuity involves a smooth transition and routing between AI chatbots and human customer service agents (Chen et al., 2022). When AI chatbots are well-integrated with fallback protocols for humans, users benefit from both channels’ strengths without experiencing service interruptions. This seamless switch ensures users receive ongoing, consistent service without having to repeat their questions or context during escalation (Chen et al., 2022; Leocádio et al., 2024). Such collaboration boosts service utility by combining AI’s speed and consistency with human empathy and problem-solving skills, increasing perceived usefulness. Additionally, Prentice et al. (2020) indicated that accessible human support offers users security and confidence. When users know that complex or sensitive issues can be smoothly escalated with their interaction history, their anxiety is reduced and satisfaction improves. Moreover, preserving interaction context during AI–human handoffs demonstrate organizational competence and respect for users’ time, thereby further enhancing satisfaction (Leocádio et al., 2024; de Oliveira Santini et al., 2025). Thus, the following hypotheses are proposed.

*H4a*: Human-AI service continuity positively influences perceived usefulness of AI chatbot services.

*H4b*: Human-AI service continuity positively influences satisfaction with AI chatbot services.

### 3.2 Effects of perceived usefulness and satisfaction on purchase intention

Perceived usefulness, as a core construct in the TAM (Davis, 1989), is expected to positively influence purchase intention. When users perceive AI chatbots as useful tools that enhance their shopping efficiency and effectiveness, they develop positive attitudes toward the e-commerce platform and are more motivated to complete purchase transactions (Rana et al., 2024). The cognitive evaluation that the AI chatbot provides functional value reduces perceived risks and barriers associated with online shopping, increasing users’ confidence and willingness to purchase (Ashfaq et al., 2020). Empirical research has consistently demonstrated the significant positive effect of perceived usefulness on behavioral intention across diverse technology contexts (Venkatesh et al., 2003; King and He, 2006).

Satisfaction is also hypothesized to positively influence purchase intentions. According to ECT, satisfied users are more likely to develop favorable attitudes and behavioral intentions toward the service provider (Oliver, 1980). Satisfaction reflects a positive cumulative evaluation of the service experience, which builds trust, reduces perceived risk, and strengthens the user’s relationship with the e-commerce platform (Møller et al., 2025). Satisfied customers are more confident in their purchase choices, are more inclined to suggest the platform to others, and are more likely to make additional purchases. Empirical studies have shown a strong link between satisfaction and purchase intention (Shahbandi, 2025). Thus, the following hypotheses are proposed:

*H5*: Perceived usefulness positively influences purchase intention.

*H6*: Satisfaction positively influences purchase intention.

### 3.3 Mediating effects of perceived usefulness and satisfaction

Perceived usefulness is expected to mediate the effects of all four dimensions of AI chatbot service quality on purchase intention. Based on the TAM (Davis, 1989; Venkatesh and Bala, 2008), perceived usefulness reflects users’ cognitive judgment of whether a technology improves their task performance and decision-making efficiency. In AI-mediated service interactions, AI chatbot service quality boosts perceived usefulness, which then drives purchase intention, reflecting the rational and utilitarian basis of consumer choice (Ashfaq et al., 2020). Chen et al. (2022) demonstrated that AI chatbot service quality, including personalization, semantic understanding, and human-AI collaboration, significantly increases customers’ perceived value and their intention to continue using the service. Ashfaq et al. (2020) further confirmed that perceived usefulness is a key predictor of users’ continuance intention toward AI-powered service agents, along with perceived enjoyment and ease of use. Existing research consistently demonstrates that when AI chatbots offer personalized recommendations and tailored assistance, users perceive the service as more useful, which in turn boosts their purchase intentions (Hsu and Lin, 2023; An and Ngo, 2025). Similarly, AI chatbots that convey social presence through human-like interactions, such as anthropomorphic cues, conversational warmth, and emotional responsiveness, may enhance users’ engagement and willingness to utilize the AI chatbot’s features, thereby elevating perceptions of the chatbot’s utility in facilitating shopping tasks (Blut et al., 2021; Cheng et al., 2022). High-quality, accurate, relevant, and comprehensive information allows AI chatbot users to make better-informed decisions with less cognitive effort, thereby boosting their perception of its usefulness (Ashfaq et al., 2020; Ameen et al., 2021). Additionally, seamless handovers from AI chatbots to human agents ensure that users receive appropriate support while maintaining context, which further increases the service system’s overall utility (Chen et al., 2022; Haugeland et al., 2022). These points collectively indicate that perceived usefulness is a key cognitive process through which each aspect of AI chatbot service quality influences purchase intention. Thus, the following hypotheses are proposed:

*H7a*: Perceived usefulness mediates the relationship between perceived personalization and purchase intention.

*H7b*: Perceived usefulness mediates the relationship between social presence and purchase intention.

*H7c*: Perceived usefulness mediates the relationship between information quality and purchase intention.

*H7d*: Perceived usefulness mediates the relationship between human-AI service continuity and purchase intention.

Satisfaction is also expected to mediate the effects of the four dimensions of AI chatbot service quality on purchase intention. From an affective perspective, these service quality dimensions may shape users’ emotional responses to AI chatbot interactions, thereby enhancing satisfaction and ultimately strengthening purchase intention. Specifically, personalized service experiences can elicit positive emotional responses and make users feel valued. This, in turn, enhances satisfaction and strengthens purchase intention (Shahzad et al., 2024; Møller et al., 2025). Similarly, AI chatbots with social presence, characterized by human warmth and a personable approach, can elicit positive affective responses that improve overall satisfaction (Deng and Yan, 2025). In addition, providing accurate and dependable information reduces user frustration and uncertainty, fostering positive service experiences that increase satisfaction (Chen Q. et al., 2023; Hsu and Lin, 2023). Moreover, ensuring smooth human backup, when needed, can build a sense of security and trust, further enhancing overall satisfaction (Li J. et al., 2023; Lin et al., 2024). Taken together, these arguments suggest an affective pathway in which AI chatbot service quality enhances satisfaction, which in turn promotes purchase intention (Akdemir and Bulut, 2024; Shahzad et al., 2024). Thus, the following hypotheses are proposed:

*H8a*: Satisfaction mediates the relationship between perceived personalization and purchase intention.

*H8b*: Satisfaction mediates the relationship between social presence and purchase intention.

*H8c*: Satisfaction mediates the relationship between information quality and purchase intention.

*H8d*: Satisfaction mediates the relationship between human-AI service continuity and purchase intention.

Figure 1 shows the research model that consolidates all the hypotheses formulated in this study. It depicts how the four dimensions of AI chatbot service quality, namely perceived personalization, social presence, information quality, and human-AI service continuity, influence purchase intention indirectly through perceived usefulness and satisfaction as dual mediators. This comprehensive model offers an integrated framework for understanding the cognitive and affective mechanisms through which AI chatbot service quality influences consumer purchase intention in e-commerce settings.

**Figure 1:**
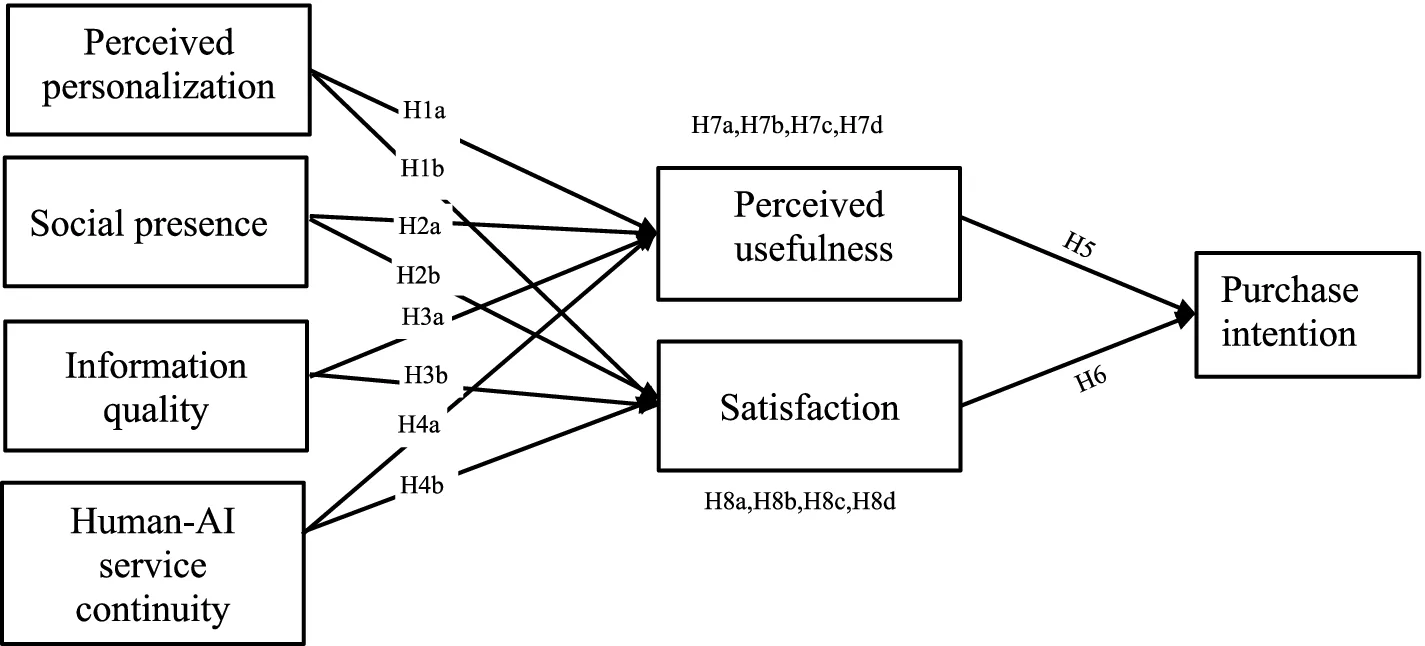
Research model and hypotheses.

## 4 Methodology

### 4.1 Data collection

The cross-sectional survey was conducted in March 2026 using an online questionnaire on the Wenjuanxing^1^ platform, targeting Taobao consumers of various age groups. To reach this diverse demographic, survey links and QR codes were widely distributed through multiple WeChat and QQ community groups. While probabilistic sampling is preferred for statistical generalizability, non-probability sampling techniques are widely recognized as appropriate and necessary when investigating specific or hard-to-reach digital user groups (Baltar and Brunet, 2012). Due to the lack of an accessible sampling frame of consumers with prior AI chatbot experience, combining convenience and snowball sampling via social media was the most practical approach to recruit qualified, information-rich respondents. A total of 620 questionnaires were distributed over 4 weeks. After excluding responses with straight-line answer patterns, incomplete data, or very short completion times (less than 1 min), 493 valid responses remained, yielding a response rate of 79.5%. This sample size exceeds the minimum required for partial least squares structural equation modeling (PLS-SEM), which necessitates at least 10 times the number of arrows pointing to a latent variable (Hair et al., 2019; Memon et al., 2020). Given the model’s complexity, a sample of 493 provided adequate statistical power to identify significant effects, even when effect sizes are small.

### 4.2 Measurement development

To ensure the validity and reliability of our measurement instruments, all construct measures were adapted from well-established scales previously validated across information systems, marketing, and human-computer interaction literature. These items were carefully modified to fit the specific context of AI chatbot services in e-commerce. Perceived personalization was measured using six items adapted from Shen et al. (2024). These items capture perceptions of the chatbot’s ability to offer tailored recommendations, understand personalized needs, analyze spending habits, provide diverse interactive features, suggest interesting products, and offer customizable services. Social presence was assessed with five items adapted from Deng and Yan (2025). These items focused on perceptions of human contact, personalness, sociability, human warmth, and human sensitivity during interactions with the AI chatbot. Information quality was measured via six items adapted from Møller et al. (2025), evaluating the sufficiency, clarity, accuracy, reliability, timeliness, and responsiveness of the information provided by the AI chatbot. Human-AI service continuity was measured using four items adapted from Chen et al. (2022). This construct was originally termed “human-AI collaboration.” In this study, it was renamed to better match the research context. The items mainly reflect seamless transfer between AI and human agents, bidirectional transition capabilities, service context preservation, and retention of interaction history to ensure uninterrupted service. Perceived usefulness was measured with five items adapted from Ashfaq et al. (2020). These items assessed whether the AI chatbot helps users complete tasks faster, functions as a helpful shopping tool, reduces task time, and improves shopping productivity. Satisfaction was measured using four items adapted from Møller et al. (2025), capturing enjoyment, conversation pleasantness, perceived value, and innovativeness of the chatbot. Purchase intention was measured using three items adapted from Pavlou (2003), measuring intentions to buy, future purchase predictions, and the likelihood of future transactions. All items were rated on a five-point Likert scale from 1 (strongly disagree) to 5 (strongly agree). A pilot study with 30 respondents was conducted before the main survey to refine item wording and ensure clarity within the Chinese e-commerce context. Based on the pilot study feedback, minor adjustments were made to improve readability and relevance.

### 4.3 Data analysis method

Data analysis was performed using partial least squares structural equation modeling (PLS-SEM) with SmartPLS 4 software. PLS-SEM was selected instead of covariance-based structural equation modeling for multiple reasons. First, this study aims to predict key target constructs (purchase intention) and identify significant driver constructs, which aligns with the prediction-oriented nature of PLS-SEM (Hair et al., 2019). Second, the research model incorporates multiple mediation effects, which can be analyzed more flexibly using PLS-SEM’s bootstrapping procedures (Hair et al., 2022). Third, PLS-SEM does not require multivariate normality assumptions and can simultaneously handle formative and reflective measurement models (Hair et al., 2019). The analysis followed the two-stage approach recommended by Hair et al. (2019): (1) assessment of the measurement model to evaluate reliability and validity, and (2) assessment of the structural model to test the hypothesized relationships. The measurement model assessment included evaluations of indicator reliability (outer loadings), internal consistency reliability (Cronbach’s alpha and composite reliability), convergent validity (average variance extracted, AVE), and discriminant validity (HTMT criterion). The structural model assessment involved evaluations of collinearity (VIF), coefficient of determination (R-squared), effect sizes (f-squared), path significance and mediation analysis.

## 5 Analysis and results

### 5.1 Demographic information

A total of 493 valid responses were gathered, and the respondents’ demographic profiles are presented in Table 1. Regarding gender distribution, the majority of respondents were female, making up 56.6% (*n* = 279), while males accounted for 43.4% (*n* = 214). Most participants were young adults, 40.2% were between 18 and 25 years old, followed by 30.8% aged 26–35. These two groups combined represented over 70% of the sample. Respondents aged 36–45, 46–55, and 56 and above constituted 17.6, 7.7, and 3.7%, respectively. Regarding education, more than half of the respondents (51.9%) held a bachelor’s degree, 18.1% had a master’s degree or higher, 19.5% had completed junior college, and 10.5% had a high school education or below. In terms of occupation, corporate employees constituted the largest group (37.6%), followed by students (26.0%), government or institution staff (18.1%), freelancers (11.0%), and others (7.3%). With respect to online shopping behavior, 6.5% (*n* = 32) shopped online less than 1 time per month, 21.9% (*n* = 108) shopped 1–2 times, 37.7% (*n* = 186) shopped 3–5 times per month, 22.7% (*n* = 112) shopped 6–10 times, and 11.2% (*n* = 55) shopped more than 10 times monthly. Regarding monthly online spending, 15.4% (*n* = 76) spent below 500 RMB, 37.9% (*n* = 187) spent between 501–1,000 RMB, 28.2% (*n* = 139) spent 1,001–1,500 RMB, 11.2% (*n* = 55) spent 1,501–2,000 RMB, and 7.3% (*n* = 36) spent above 2,000 RMB.

**Table 1:** Demographic profile of respondents (*N* = 493).

Variable	Category	*n*	%
Gender	Female	279	56.6
	male	214	43.4
Age	18–25 years old	198	40.2
	26–35 years old	152	30.8
	36–45 years old	87	17.6
	46–55 years old	38	7.7
	56 years old and above	18	3.7
Education	High school and below	52	10.5
	Junior college	96	19.5
	Bachelor’s degree	256	51.9
	Master’s degree or above	89	18.1
Occupation	Student	128	26.0
	Corporate employee	186	37.6
	Government or institution staff	89	18.1
	Freelancer	54	11.0
	Other	36	7.3
Monthly online shopping frequency	Less than 1 time	32	6.5
	1–2 times	108	21.9
	3–5 times	186	37.7
	6–10 times	112	22.7
	More than 10 times	55	11.2
Monthly online spending (RMB)	Below 500 yuan	76	15.4
	501–1,000 yuan	187	37.9
	1,001–1,500 yuan	139	28.2
	1,501–2,000 yuan	55	11.2
	Above 2,000 yuan	36	7.3

### 5.2 Measurement model assessment

To prevent potential common method bias from using cross-sectional, self-reported survey data, we performed Harman’s single-factor test before evaluating the measurement model (Podsakoff et al., 2003). We subjected all measurement items of the main constructs to an unrotated principal component analysis. The results showed that the first and largest unrotated factor explained only 26.085% of the total variance. This value was far below the commonly used threshold of 50%. Therefore, no single factor appeared to dominate the covariance among the measures. Common method bias was unlikely to be a major concern in this study.

The measurement model was tested to assess the reliability and validity of the seven reflective constructs. Following the two-step approach proposed by Hair et al. (2019), we first analyzed the measurement model before assessing the structural model. Internal consistency was evaluated through Cronbach’s alpha (α) and composite reliability. As shown in Table 2, the Cronbach’s alpha values ranged from 0.875 to 0.925, and the composite reliability values ranged from 0.878 to 0.929, all exceeding the recommended threshold of 0.70 (Hair et al., 2022), indicating satisfactory internal consistency for all constructs. Convergent validity was confirmed through standardized factor loadings and AVE. All factor loadings were above the conservative threshold of 0.70, ranging from 0.775 to 0.912. Additionally, all AVE values fell between 0.673 to 0.801, exceeding the minimum cutoff of 0.50 (Fornell and Larcker, 1981). These findings show that the measurement model has sufficient reliability and convergent validity. Therefore, it is suitable for further structural model testing.

**Table 2:** Reliability and validity of the research model.

Constructs	Items	Outer loading	Cronbach α	CR	AVE
Purchase Intention (PI)	PI1	0.912	0.875	0.878	0.801
	PI2	0.881			
	PI3	0.891			
Perceived Usefulness (PU)	PU1	0.888	0.921	0.923	0.760
	PU2	0.865			
	PU3	0.879			
	PU4	0.869			
	PU5	0.858			
Satisfaction (SA)	SA1	0.883	0.897	0.898	0.764
	SA2	0.854			
	SA3	0.898			
	SA4	0.860			
Perceived Personalization (PP)	PP1	0.799	0.912	0.920	0.694
	PP2	0.876			
	PP3	0.805			
	PP4	0.881			
	PP5	0.852			
	PP6	0.781			
Information Quality (IQ)	IQ1	0.867	0.925	0.929	0.727
	IQ2	0.830			
	IQ3	0.892			
	IQ4	0.850			
	IQ5	0.817			
	IQ6	0.859			
Social Presence (SP)	SP1	0.804	0.879	0.891	0.673
	SP2	0.809			
	SP3	0.858			
	SP4	0.775			
	SP5	0.852			
Human-AI service continuity (HASC)	HASC1	0.879	0.899	0.905	0.768
	HASC2	0.866			
	HASC3	0.895			
	HASC4	0.865			

Discriminant validity was evaluated using the heterotrait-monotrait (HTMT) ratio of correlations. According to the conservative threshold suggested by Henseler et al. (2015), all HTMT values should be below 0.85 to confirm discriminant validity. As shown in Table 3, the HTMT ratios ranged from 0.042 to 0.767, with all values satisfying this criterion. This evidence indicates that each construct captures a distinct phenomenon. It is separate from the other constructs in the model. Therefore, the results confirm adequate discriminant validity for all seven variables.

**Table 3:** Discriminant validity (HTMT).

Construct	HASC	IQ	PI	PP	PU	SA	SP
HASC							
IQ	0.048						
PI	0.198	0.413					
PP	0.135	0.056	0.227				
PU	0.103	0.335	0.715	0.093			
SA	0.219	0.495	0.767	0.262	0.556		
SP	0.048	0.045	0.204	0.042	0.303	0.052	

### 5.3 Structural model assessment

Table 4 presents the structural model results in terms of the coefficient of determination (R^2^), adjusted R^2^, and Stone–Geisser’s Q^2^ for the three endogenous constructs. The model demonstrates moderate explanatory power for purchase intention (R^2^ = 0.585) and satisfaction (R^2^ = 0.333), while the explanatory power for perceived usefulness (R^2^ = 0.196) is relatively weak, based on the conventional thresholds of 0.25, 0.50, and 0.75, as weak, moderate, and substantial levels, respectively (Henseler and Sarstedt, 2013; Hair et al., 2019). Regarding predictive relevance, the Stone-Geisser’s Q^2^ values derived from the blindfolding procedure were 0.462 for purchase intention, 0.249 for satisfaction, and 0.146 for perceived usefulness, all exceeding the critical value of zero, thereby confirming the model’s predictive capability for these endogenous variables (Geisser, 1974). In addition, the VIF was employed to assess multicollinearity among items. The VIF values ranged from 1.000 to 1.344, indicating no multicollinearity issues.

**Table 4:** R^2^, adjusted R^2^, and Q^2^ results.

Endogenous variables	R^2^	R^2^_adj_	Q^2^
Purchase intention	0.585	0.583	0.462
Perceived usefulness	0.196	0.190	0.146
Satisfaction	0.333	0.328	0.249

In accordance with Hair et al. (2019), all hypotheses were assessed using a 5,000 bootstrap-sample procedure with one-tailed tests. The PLS-bootstrapping results are illustrated in Figure 2. Table 5 shows that perceived personalization had a significant positive effect on perceived usefulness (*β* = 0.112, *p* = 0.008) and satisfaction (*β* = 0.286, *p* < 0.001), thus supporting H1a and H1b. Social presence had a significant positive effect on perceived usefulness (*β* = 0.275, *p* < 0.001); however, its effect on satisfaction was not significant (*β* = 0.044, *p* = 0.201), so H2a was supported, whereas H2b was not. Information quality had a significant positive effect on both perceived usefulness (*β* = 0.317, *p* < 0.001) and satisfaction (*β* = 0.471, *p* < 0.001), thus supporting H3a and H3b. Human-AI service continuity had a significant positive influence on perceived usefulness (*β* = 0.119, *p* = 0.005) and satisfaction (*β* = 0.249, *p* < 0.001), supporting H4a and H4b. Furthermore, perceived usefulness significantly and positively influenced purchase intention (*β* = 0.403, *p* < 0.001), and satisfaction significantly and positively influenced purchase intention (*β* = 0.477, *p* < 0.001), thus H5 and H6 were supported. In summary, H1a, H1b, H2a, H3a, H3b, H4a, H4b, H5, and H6 were supported, while H2b was not supported.

**Figure 2:**
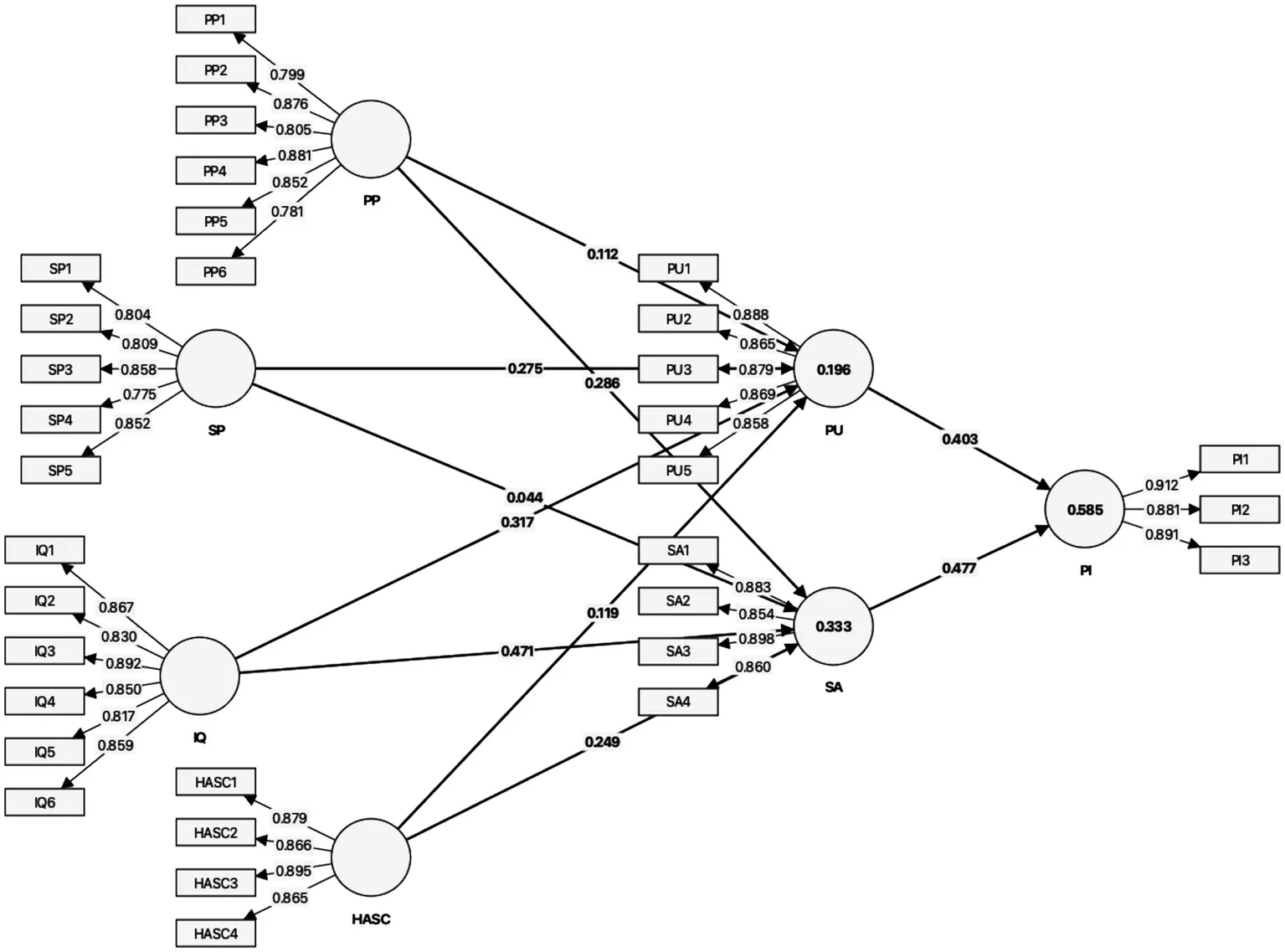
Structural model results.

**Table 5:** Results of the path analysis.

Hypothesis	Path	*β*	S. E.	*T*-values	*p*-values	*f^2^*	Decision
H1a	PP → PU	0.112	0.042	2.649	0.008	0.015	Supported
H1b	PP → SA	0.286	0.042	6.888	0.000	0.121	Supported
H2a	SP → PU	0.275	0.038	7.212	0.000	0.094	Supported
H2b	SP → SA	0.044	0.035	1.278	0.201	0.003	Not Supported
H3a	IQ → PU	0.317	0.039	8.139	0.000	0.124	Supported
H3b	IQ → SA	0.471	0.035	13.508	0.000	0.332	Supported
H4a	HASC → PU	0.119	0.043	2.804	0.005	0.017	Supported
H4b	HASC → SA	0.249	0.040	6.295	0.000	0.091	Supported
H5	PU → PI	0.403	0.036	11.220	0.000	0.291	Supported
H6	SA → PI	0.477	0.036	13.087	0.000	0.408	Supported

### 5.4 Mediation analysis

In this study, perceived usefulness and satisfaction were considered as mediating effects. The mediation effect was assessed using the bootstrap method proposed by Preacher and Hayes (2008), which is endorsed by Hair et al. (2022) as a suitable approach for PLS-SEM. The bootstrap procedure with 5,000 resamples was used to generate bias-corrected and accelerated 95% confidence intervals for the indirect effects. An indirect effect was considered statistically significant when the 95% confidence interval did not include zero and the *p*-value was below 0.05 (Preacher and Hayes, 2008). As shown in Table 6, perceived usefulness serves as a significant mediator in the relationship between perceived personalization and purchase intention (H7a: *β* = 0.045, *p* = 0.012), social presence and purchase intention (H7b: *β* = 0.111, *p* < 0.001), information quality and purchase intention (H7c: *β* = 0.128, *p* < 0.001), and human-AI service continuity and purchase intention (H7d: *β* = 0.048, *p* = 0.007). Therefore, H7a, H7b, H7c, and H7d are supported. Furthermore, satisfaction was found to be a significant mediator in the relationships between perceived personalization and purchase intention (H8a: *β* = 0.136, *p* < 0.001), information quality and purchase intention (H8c: *β* = 0.225, *p* < 0.001), and between human-AI service continuity and purchase intention (H8d: *β* = 0.119, *p* < 0.001). However, the indirect effect of social presence on purchase intention through satisfaction was not significant (H8b: *β* = 0.021, *p* = 0.205). Therefore, hypotheses H8a, H8c, and H8d are supported, while H8b is unsupported.

**Table 6:** Results of mediation analysis.

Hypothesis	Path	*β*	S. E.	*T*-values	*p*-values	BCI LL	BCI UL	Decision
H7a	PP → PU → PI	0.045	0.018	2.526	0.012	0.012	0.083	Supported
H7b	SP → PU → PI	0.111	0.019	5.835	0.000	0.076	0.149	Supported
H7c	IQ → PU → PI	0.128	0.020	6.421	0.000	0.090	0.168	Supported
H7d	HASC → PU → PI	0.048	0.018	2.710	0.007	0.014	0.083	Supported
H8a	PP → SA → PI	0.136	0.024	5.677	0.000	0.090	0.184	Supported
H8b	SP → SA → PI	0.021	0.017	1.268	0.205	−0.012	0.053	Not Supported
H8c	IQ → SA → PI	0.225	0.025	9.152	0.000	0.178	0.273	Supported
H8d	HASC → SA → PI	0.119	0.022	5.511	0.000	0.079	0.163	Supported

## 6 Discussion

### 6.1 Key findings

This study investigated the impact of AI chatbot service quality on consumers’ purchase intentions in e-commerce. It used a comprehensive theoretical framework that integrates ECT (Oliver, 1980), DeLone and McLean’s ISSM (DeLone and McLean, 1992), and the TAM (Davis, 1989). The analysis was based on data from 493 Chinese consumers with prior experience using AI chatbots on e-commerce platforms. The findings contribute to both the theoretical understanding and practical application of AI-mediated customer service. The empirical analysis identified four key dimensions of AI chatbot service quality, namely, perceived personalization, social presence, information quality, and human-AI service continuity. The results revealed that all four factors significantly affected perceived usefulness. Additionally, perceived personalization, information quality, and human-AI service continuity also positively impacted satisfaction. Interestingly, social presence did not have a significant direct effect on satisfaction. This suggests that in utilitarian e-commerce settings, users may value functional features more than the social and emotional aspects of AI chatbot interactions. This pattern aligns with recent studies showing that consumers value task efficiency over social engagement with AI service agents (Hsu and Lin, 2023; Gao et al., 2025).

Among the four dimensions of AI chatbot service quality, information quality was the most influential factor in predicting both perceived usefulness and satisfaction. This aligns with the core principle of the ISSM, which states that information quality is a fundamental aspect of system success (DeLone and McLean, 1992, 2003), and it extends this concept to the realm of AI chatbots. The empirical evidence highlights that consumers assess AI chatbot service quality based on the accuracy, completeness, timeliness, and reliability of the information they deliver. This finding supports recent research suggesting that information quality is a key factor in building user trust and influencing behavioral intentions in AI-based service interactions (Luo et al., 2019; Chen et al., 2022).

In terms of the direct predictors of purchase intention, both perceived usefulness and satisfaction showed significant positive effects. Between these two mediating variables, satisfaction had a stronger direct effect on purchase intention than perceived usefulness. The mediation analysis also showed that perceived usefulness mediated the effects of all four service quality dimensions on purchase intention, whereas satisfaction mediated the effects of perceived personalization, information quality, and human-AI service continuity, but not the effect of social presence.

The unsupported hypothesis regarding social presence’s impact on satisfaction and the lack of a significant mediation effect through satisfaction deserve careful consideration. This result differs from findings in hedonic service contexts, where social presence has been shown to significantly enhance user satisfaction (Konya-Baumbach et al., 2023; Deng and Yan, 2025). However, it aligns with recent findings suggesting that in task-focused e-commerce settings, consumers tend to prioritize functional benefits over social or emotional connections when assessing AI chatbot service quality (Ruan and Mezei, 2022; Hsu and Lin, 2023). The significant effect of social presence on perceived usefulness, however, indicates that human-like interaction aspects do contribute to consumers’ cognitive evaluations of AI chatbot utility, even if they do not directly translate into affective satisfaction.

### 6.2 Theoretical implications

This study’s findings contribute valuable insights to the literature on electronic commerce and information systems. First, this study advances understanding of AI chatbot service quality by combining multidimensional service attributes with a dual-mediation framework. This framework is based on ECT (Oliver, 1980), the ISSM (DeLone and McLean, 1992, 2003), and TAM (Davis, 1989). Although these AI chatbot service quality dimensions, perceived usefulness, and satisfaction are well-established in information systems and marketing research, past studies focused on either cognitive utility (TAM) or affective evaluation (ECT). Integrating them into a unified model, this research offers a more comprehensive account of consumer decision-making in non-human service interactions. It shows that AI chatbot service quality influences purchase intention through distinct psychological pathways, highlighting how functional and selected relational aspects shape purchase intention through different psychological.

Second, this study advances the emerging literature on AI chatbot service quality by operationalizing and validating four key dimensions, namely, perceived personalization, social presence, information quality, and human-AI service continuity. These are analyzed within a single empirical framework. Although recent research has contributed valuable frameworks for AI chatbot service quality (Chen Q. et al., 2023; Shen et al., 2024), empirical studies exploring how these dimensions influence downstream consumer outcomes are limited. In addition, some recent studies have treated AI chatbot service quality as a higher-order construct (Leong et al., 2026; Sharma and Tandon, 2026). This approach may make it difficult to identify which dimensions exert stronger effects on purchase intention. In contrast, this study treats the four dimensions as separate exogenous variables. This design allows a clearer examination of their distinct effects on perceived usefulness, satisfaction, and purchase intention. By doing so, the study offers more detailed insights into the relative importance of each dimension and further enhances the theoretical understanding of AI chatbot service quality.

Third, the differing impacts of social presence on perceived usefulness versus satisfaction contribute to the ongoing theoretical debate about the role of anthropomorphism and social cues in human-AI interactions (Blut et al., 2021; Konya-Baumbach et al., 2023). Social presence significantly affects perceived usefulness but not satisfaction in an e-commerce setting. This result indicates that the effect of social presence may depend on context and differ between hedonic and utilitarian services. This contextual dependence offers valuable insights into the limits of social presence theory in AI-mediated services. It also emphasizes the need for more detailed frameworks that clarify when and how social presence affects various user outcomes.

Finally, the study empirically confirms two mediation pathways, namely, the cognitive pathway through perceived usefulness and the affective pathway through satisfaction, and extends the theoretical understanding of how AI chatbot service quality influences behavioral intentions in technology-mediated settings. While the TAM highlights cognitive evaluations (Davis, 1989), and ECT emphasizes satisfaction as an affective and evaluative response (Oliver, 1980), this research shows that both pathways operate in the model, although the affective pathway through satisfaction is not supported for social presence. Notably, when the two mediators are compared, satisfaction has a stronger direct impact on purchase intention than perceived usefulness. This supports the idea that consumer decision-making in AI-driven service encounters is shaped by both rational assessments and emotional responses (Bilgihan et al., 2025; Avcılar and Yenilmez, 2026), providing a more comprehensive account than either mechanism alone.

### 6.3 Practical implications

This study’s results offer practical guidance for e-commerce platform managers, AI chatbot developers, and digital marketing professionals. Since information quality significantly influences perceived usefulness and satisfaction, platforms should invest in improving the accuracy, completeness, timeliness, and reliability of their AI chatbots’ information. They can do this by deploying strong knowledge management systems, maintaining real-time updates with inventory and pricing data, and implementing advanced natural language processing algorithms that accurately interpret consumer queries and provide contextually relevant responses (Lin et al., 2024). Additionally, platforms should develop systematic quality assurance processes to monitor and enhance the chatbot’s information output continually.

Moreover, the significant effects of perceived personalization on both mediators underscore the commercial value of AI-driven personalization capabilities. E-commerce platforms should utilize consumer data analytics and machine learning algorithms to offer personalized service experiences, such as customized product recommendations, tailored interaction styles, and context-aware support based on browsing history, purchase behavior, and user preferences. Nonetheless, practitioners need to remain cautious about privacy issues that can emerge from over-personalization, as previous studies have highlighted the risk of AI personalization feeling intrusive to consumers (Shoukat et al., 2024).

Beyond personalization, the significant positive impacts of human-AI service continuity on perceived usefulness and satisfaction emphasize the need for seamless service transitions between AI chatbots and human agents. E-commerce platforms should adopt intelligent escalation strategies. These strategies can support smooth handoffs when user queries exceed the AI chatbot’s capabilities, maintain context, and prevent customers from repeating questions (Vassilakopoulou et al., 2022; Jeong and Lee, 2025). Preserving interaction history during AI-human handovers is particularly important. It reflects organizational competence and respect for users’ time, consequently increasing both functional usefulness and overall satisfaction (Leocádio et al., 2024).

Regarding social presence, the finding shows it significantly impacts perceived usefulness but not satisfaction. This indicates that practitioners should tailor AI chatbot social features primarily to improve task-related usefulness rather than emotional engagement alone. Social presence interaction qualities, such as conversational warmth, polite language, and human-like responsiveness, should be used to facilitate information sharing and task completion, not just for their own sake (Cheng et al., 2022; Casaló et al., 2025). This utilitarian orientation toward social presence design is especially important in e-commerce customer service, where customers prefer quick resolutions to transactional queries over social interaction (Hsu and Lin, 2023).

Both perceived usefulness and satisfaction serve as key mediators, with satisfaction having a more direct impact on purchase intention. As a result, e-commerce managers should implement a dual-pathway strategy for AI chatbot service enhancement. While improving functional utility is essential for increasing perceived usefulness, creating positive overall user experiences may generate greater purchase conversion returns. These experiences should include reliable service, engaging interaction design, and consistent performance that meets or surpasses expectations (Bilgihan et al., 2025). This approach indicates that strategies should focus simultaneously on boosting cognitive utility enhancement and affective experience in an integrated way.

### 6.4 Limitations and future research

Although this study offers valuable theoretical and practical insights, it has certain limitations that highlight areas for future investigation. The use of a cross-sectional research design constrains the ability to determine causality among the variables. While PLS-SEM analysis sheds light on the directional links proposed in the theoretical framework, the temporally bounded nature of cross-sectional data prevents firm causal conclusions. Future studies should adopt longitudinal approaches. These approaches can track changes in consumers’ perceptions of AI chatbot service quality, perceived usefulness, satisfaction, and purchase intention over time. They may also support stronger causal conclusions and provide a clearer understanding of how these relationships evolve.

Second, relying on self-reported survey data can introduce potential common method bias. However, Harman’s single-factor test results suggest that this bias is not a significant concern in our dataset. The measurement model also showed good reliability and validity, and the HTMT criterion for discriminant validity was met. To further address this issue, future research could include objective measures of purchase intention, like actual purchase records, clickstream data, or transaction logs, and gather data from multiple sources. These multi-method approaches would strengthen the findings and lessen the impact of self-report bias.

Third, this study examined the Chinese e-commerce ecosystem, focusing on consumers’ interactions with AI chatbots on Taobao. As one of the world’s largest and most technologically advanced markets, the Chinese e-commerce sector’s results may not fully apply to other platforms, industries, or countries with different technological frameworks and consumer habits. Additionally, the reliance on convenience and snowball sampling methods is a methodological limitation. Although these approaches were necessary to target our specific audience, they introduce potential selection bias and limit the generalizability of the findings to the wider consumer base. Future research should explore other e-commerce platforms like JD.com, Pinduoduo, Amazon, and eBay, as well as service sectors such as banking, tourism, and healthcare. It should also consider different cultural contexts and employ probabilistic or stratified sampling techniques to verify results across diverse demographic groups.

Fourth, this research focused on four specific dimensions of AI chatbot service quality. However, it did not include all factors that may influence consumer purchase intention. Emerging service quality aspects like perceived empathy, ethical AI practices, transparency in algorithmic decisions, and data privacy might also significantly influence how consumers perceive and interact with AI chatbots. Future studies could enhance the AI chatbot service quality framework by adding these factors and exploring their combined impact alongside the four dimensions investigated in this study.

## 7 Conclusion

This study examines how AI-chatbot service quality affects consumers’ purchase intention in e-commerce by integrating ECT, the ISSM, and the TAM. Based on survey responses from 493 Chinese consumers, the findings show that perceived personalization, social presence, information quality, and human-AI service continuity significantly enhanced perceived usefulness. Perceived personalization, information quality, and human-AI service continuity also positively affected satisfaction. Among these factors, information quality was the strongest predictor. However, social presence did not have a significant direct effect on satisfaction. Both perceived usefulness and satisfaction significantly increased purchase intention, with satisfaction showing a stronger direct effect. The mediation results further indicate that perceived usefulness mediated the effects of all four service quality dimensions on purchase intention. Satisfaction mediated the effects of perceived personalization, information quality, and human-AI service continuity, but not the effect of social presence. These findings clarify the cognitive and affective pathways through which AI chatbot service quality dimensions shape purchase intention.

## Data Availability

The raw data supporting the conclusions of this article will be made available by the authors, without undue reservation.

## Footnotes

1
www.wjx.cn
