# Supplementary material for: The impact of AI chatbot service quality on consumer purchase intention: the cognitive and affective mediating mechanisms
Source: Front Psychol. 2026 Sep 29;17:1931206. doi: 10.3389/fpsyg.2026.1931206 (PMC13658087; doi:10.3389/fpsyg.2026.1931206)
Supplement: Supplementary file 1 [file Supplementary_file_1.docx]

**Appendix I: Measurement Items and Sources**

| **Variable** | **Measurement Items** | **References** |
| --- | --- | --- |
| Perceived Personalization | I can always use this store's AI chatbot to easily search for or match the specific items I want.  This store's AI chatbot can understand and provide answers that meet my personalized needs.  The AI chatbot of this store can accurately analyze my spending habits to offer customized shopping advice.  The AI chatbot of this store offers diverse interactive features (e.g., image search, voice input) to suit my habits.  The products and promotional info recommended by this store's AI chatbot are items I am genuinely interested in.  I can customize or choose the specific service options provided by this store's AI chatbot. | Shen et al. (2024) |
| Social Presence | There is a sense of human contact in this store's AI chatbot.  There is a sense of personalness in this store's AI chatbot.  There is a sense of sociability in this store's AI chatbot.  There is a sense of human warmth in this store's AI chatbot.  There is a sense of human sensitivity in this store's AI chatbot. | Deng and Yan (2025) |
| Information Quality | The AI chatbot of this store provides sufficient information.  Information provided by this store's AI chatbot is clear.  Information provided by this store's AI chatbot is accurate.  Information provided by this store's AI chatbot is reliable.  Information provided by this store's AI chatbot is up-to-date.  This store's AI chatbot provides the information I need in a timely manner. | Møller et al. (2024) |
| Human-AI Service Continuity | When this store's AI chatbot cannot solve my problem, I can easily switch to a human customer service agent.  The AI chatbot and human customer service of this store can easily switch between each other.  When I am transferred from the AI chatbot to a human agent, I don't need to repeat my questions.  The store's AI chatbot system can remember my previous questions and service history. | Chen et al. (2022) |
| Perceived Usefulness | This store's AI chatbot helps me accomplish things more quickly than a human would.  This store's AI chatbot is a useful tool for my shopping.  Using this store's AI chatbot enables me to reduce the task completion time.  Using this store's AI chatbot allows me to be more productive in my shopping.  Using this store's AI chatbot helps me to perform shopping tasks more conveniently. | Ashfaq et al. (2020) |
| Satisfaction | I enjoy using this store's AI chatbot.  It was pleasant to have a conversation with this store's AI chatbot.  When I am using this store's AI chatbot, I feel it's a valuable tool for me.  I think this store's AI chatbot is an innovative way of providing service. | Møller et al. (2024) |
| Purchase Intention | Given the chance, I intend to buy products from this store.  Given the chance, I predict that I will purchase products from this store in the future.  It is likely that I will transact with this store in the near future. | Pavlou (2003) |
